# Dysregulated complement activation during acute myocardial infarction leads to endothelial glycocalyx degradation and endothelial dysfunction via the C5a:C5a-Receptor1 axis

**DOI:** 10.3389/fimmu.2024.1426526

**Published:** 2024-07-11

**Authors:** Carl Vahldieck, Samuel Löning, Constantin Hamacher, Benedikt Fels, Bettina Rudzewski, Laura Nickel, Joachim Weil, Henry Nording, Lasse Baron, Marie Kleingarn, Christian Marcel Karsten, Kristina Kusche-Vihrog

**Affiliations:** ^1^ Department of Anesthesiology and Intensive Care Medicine, University Medical Centre Schleswig-Holstein Campus Luebeck, Luebeck, Germany; ^2^ Institute of Physiology, University of Luebeck, Luebeck, Germany; ^3^ DZHK (German Research Centre for Cardiovascular Research), Partner Site Hamburg/Luebeck/Kiel, Luebeck, Germany; ^4^ Medizinische Klinik II, Sana Kliniken Luebeck, Luebeck, Germany; ^5^ Cardioimmunology Group, Medical Clinic II, University Heart Center Luebeck, Luebeck, Germany; ^6^ Institute for Systemic Inflammation Research (ISEF), University of Luebeck, Luebeck, Germany

**Keywords:** endothelial glycocalyx, endothelial dysfunction, complement system, C5a, C5a receptor 1, myocardial infarction, nitric oxide, atomic force microscopy

## Abstract

**Introduction:**

Complement-mediated damage to the myocardium during acute myocardial infarction (AMI), particularly the late components of the terminal pathway (C5-convertase and C5b-9), have previously been characterized. Unfortunately, only few studies have reported a direct association between dysregulated complement activation and endothelial function. Hence, little attention has been paid to the role of the anaphylatoxin C5a. The endothelial glycocalyx (eGC) together with the cellular actin cortex provide a vasoprotective barrier against chronic vascular inflammation. Changes in their nanomechanical properties (stiffness and height) are recognized as hallmarks of endothelial dysfunction as they correlate with the bioavailability of vasoactive substances, such as nitric oxide (NO). Here, we determined how the C5a:C5aR1 axis affects the eGC and endothelial function in AMI.

**Methods:**

Samples of fifty-five patients with ST-elevation myocardial infarction (STEMI) vs. healthy controls were analyzed in this study. eGC components and C5a levels were determined via ELISA; NO levels were quantified chemiluminescence-based. Endothelial cells were stimulated with C5a or patient sera (with/without C5a-receptor1 antagonist “PMX53”) and the nanomechanical properties of eGC quantified using the atomic force microscopy (AFM)-based nanoindentation technique. To measure actin cytoskeletal tension regulator activation (RhoA and Rac1) G-LISA assays were applied. Vascular inflammation was examined by quantifying monocyte-endothelium interaction via AFM-based single-cell-force spectroscopy.

**Results:**

Serum concentrations of eGC components and C5a were significantly increased during STEMI. Serum and solely C5a stimulation decreased eGC height and stiffness, indicating shedding of the eGC. C5a enhanced RhoA activation, resulting in increased cortical stiffness with subsequent reduction in NO concentrations. Monocyte adhesion to the endothelium was enhanced after both C5a and stimulation with STEMI serum. eGC degradation- and RhoA-induced cortical stiffening with subsequent endothelial dysfunction were attenuated after administering PMX53.

**Conclusion:**

This study demonstrates that dysregulated C5a activation during AMI results in eGC damage with subsequent endothelial dysfunction and reduced NO bioavailability, indicating progressively developing vascular inflammation. This could be prevented by antagonizing C5aR1, highlighting the role of the C5a:C5a-Receptor1 axis in vascular inflammation development and endothelial dysfunction in AMI, offering new therapeutic approaches for future investigations.

## 1 Introduction

During acute myocardial infarction (AMI) several innate immune pathways, including those of the complement system, are activated in the early steps of the inflammatory response to myocardial ischemia (1). There are three major pathways of complement activation: the classical, lectin, and alternative pathways. The classical pathway is initiated by the binding of C1q to immune complexes or other activating molecules like pentraxins (2). The lectin pathway is activated by recognition of carbohydrate patterns by ficolins or mannose-binding lectin (MBL) (2–4). The alternative pathway is triggered by spontaneous hydrolyzation of C3, amplifying the formation and deposition of C3b (4). All three pathways converge at the cleavage of C3 into C3a and C3b, leading to the formation of the C5 convertase (C4bC2bC3b or C3bBbC3b) that cleaves C5 into C5a and C5b (4). When C5b associates with C6 and C7, the complex becomes inserted into cell membranes and interacts with C8, inducing the binding of several units of C9 to form a lytic pore, the terminal complement complex (C5b-9n) also known as membrane attack complex (4).

Dysregulated activation of the complement system during AMI has been shown to be an important mediator of inflammatory damage and is associated with larger infarctions and poor clinical outcomes (5). C3 breakdown products and leukocyte infiltration have been demonstrated in infarcted myocardium highlighting the critical role of the complement cascade in triggering inflammation in the ischemic myocardium (6) The role of complement as a mediator of myocardial inflammation has been investigated by quantifying the products of complement activation, C3b, C4b, Bb and C5b-9n, in patients with AMI. Although serum elevation of the early complement pathway’s components like C1r, C3 and Factor B during AMI was demonstrated (7) only the late components (C5 convertase and C5b-9) of the complement pathway were correlated with necrotic mass of the myocardium and Troponin-T levels. Furthermore, C5a and C5b-9 have been shown to increased polymorphonuclear leukocytes adherence and directly induce myocardial injury (8).

Particularly, the mechanisms underlying complement-mediated injury to the myocardium mediated by the late components of the complement system’s terminal pathway have previously been characterized (9–11). However, dysregulated complement activation causes not only myocardial damage, but also damage to the vascular endothelium (12, 13). Although the terminal pathway seems to play an important role in the development of endothelial damage during dysregulated complement activation, little attention has been paid to the effect of the anaphylatoxin C5a (14). Unfortunately, only few data are available so far on the mechanisms underlying C5a-induced damage to the vascular endothelium and especially the development of chronic vascular inflammation.

C5a is considered the most potent proinflammatory anaphylatoxin (5). It shows high proinflammatory activity and induces activation and polarization of lymphocytes and increased leukocyte adherence to endothelial cells (15, 16). Plasma concentrations of C5a can rise several fold under pathophysiological conditions such as AMI, causing local increases in blood flow, smooth muscle contraction, edema, cytokine storm, mast cell degranulation, and increased vascular permeability (1, 5, 13, 15, 17). The influence of the anaphylatoxin C5a on the endothelium in the context of AMI has been underestimated to date and mechanistic knowledge of C5a-mediated changes both in endothelial cells and on the endothelial surface is limited.

During AMI the endothelium becomes an activated phenotype, resulting in a proinflammatory and prothrombotic state (18). Under healthy conditions, endothelial cells form a continuous layer along the entire vasculature, representing a crucial interface between blood and tissue. The endothelium is able to respond to external stimuli by secreting vasoactive substances and endothelium-derived relaxing factors such as nitric oxide (NO) to regulate homeostasis within the vascular system (19, 20). Healthy endothelium is covered by a negatively charged, brush-like layer: the endothelial glycocalyx (eGC). The eGC is a multifunctional layer of membrane-bound, carbohydrate-rich molecules, mostly consisting of glycoproteins and proteoglycans. Together with the underlying cellular cortex, an actin-rich layer 50–150 nm beneath the plasma membrane, the eGC forms a functional compartment that enables endothelial cells to detect and respond to external stimuli (20). Changes in the nanomechanical properties (such as stiffness) inversely correlate with endothelial NO release. Increased stiffness of the cellular actin cortex therefore reduces NO production and vice versa (20). Thus, the cellular actin cortex and the eGC can be seen as key vasoprotective players.

RhoA and its downstream effector, Rho-dependent coiled-coil kinase (ROCK), belong to the GTPase members of the Rho subfamily (21). Together, the RhoA/ROCK pathway regulates a wide array of cellular functions, including cellular polarity, motility, adhesion, proliferation, contraction, and migration (22). This signaling pathway is also known to regulate cell contraction, and permeability function in endothelial cells (22). Also, the cytoskeletal dynamics, such as changes in cell stiffness through actin polymerization/depolymerization, are regulated via the RhoA/ROCK pathway and dysregulation of this pathway has been implicated in various vascular disorders (21–23). Although reperfusion of the hypoxic myocardial tissue after AMI is critical for reoxygenation and organ salvage, it also results in myocardial ischemia and reperfusion (I/R) injury, causing further damage to the reperfused myocardial tissue (13). In fact, the vascular endothelium, or the eGC, is also impaired in the context of I/R (18). Degradation of the eGC is furthermore considered a hallmark in I/R-related endothelial dysfunction, which further impairs local microcirculation with a feed-forward loop of organ damage due to vasoconstriction, leukocyte adherence, and activation of the immune response, including the complement cascade (1, 18).

The aim of this study was to evaluate the effects of the complement anaphylatoxin C5a on the nanomechanical properties of the eGC and cellular cortex in the context of AMI. In the present study we (i) determine the importance of AMI-induced C5a elevation on mechanical changes to the endothelial cell surface using the atomic force microscopy (AFM)-based nanoindentation technique, (ii) evaluate the contribution of C5a to endothelial dysfunction and the condition of the eGC and to leukocyte-endothelium interaction, and (iii) investigate the effect of administering the C5a-Receptor-1 antagonist “PMX53” on restoring vascular nanomechanics and endothelial function.

## 2 Methods

### 2.1 Study population

Fifty-five patients with a first onset of ST-elevation myocardial infarction (STEMI) were recruited at the University of Luebeck in cooperation with the Department of Cardiology and Angiology of the Sana-Kliniken-Luebeck hospital (Germany) in accordance with the Declaration of Helsinki and approved by the Local Ethics Committee (Case: 19–310). Patients with STEMI who received a percutaneous coronary intervention (PCI) as first-line therapy during the first 120 min after STEMI was diagnosed were enrolled randomly after obtaining written informed consent. Serum samples were collected during emergency PCI (STEMI group). Fifty-five age- and sex-matched volunteers without cardiovascular comorbidities served as controls (CTR group). Serum samples from patients and controls were immediately treated according to Brandwijk et al. (24), who recommended assessing individual complement components in whole blood. Therefore, samples were kept on ice and centrifuged at 4°C within 60 min after collection. Thereafter, the samples were snap frozen and stored at -80°C. Patients in whom cardiopulmonary resuscitation was performed were excluded, as were patients who died during or after PCI. Further exclusion criteria were age below 18 years, pregnancy, or consent not given.

### 2.2 Enzyme-linked immunosorbent assay

Concentrations of the complement anaphylatoxin C5a and of soluble glycocalyx constituents (syndecan-1, heparan sulfate, and hyaluronan) were quantified by enzyme-linked immunosorbent assay (ELISA) (C5a: Complement C5a Human ELISA Kit; Thermo Fisher Scientific, Hamburg, Germany; catalog: BMS2088/Syndecan-1: Human CD138 ELISA kit, Diaclone Research, Besançon, France; catalog: 950.640.192/Heparan Sulfate: Human Heparan sulfate Proteoglycan (HSPG) ELISA Kit, MBS, San Diego, CA, USA; catalog: MBS2023323/Hyaluronan: Hyaluronan Quantikine ELISA Kit, R&D Systems, Minneapolis, MN, USA; catalog: DHYAL0).

### 2.3 Nitric oxide measurements

Concentrations of NO products [nitrites (NO_2_^−^) and nitrates (NO_3_^−^)] were determined using the chemiluminescence detector Sievers Nitric-Oxide Analyzer 280-i (NOA-280i; GE Water & Process Technologies, Analytic Instruments; Boulder, CO, USA). The assay is based on the reduction of all nitrates and nitrites into NO by vanadium(III)-chloride (VCl_3_). Briefly, NO reacts with ozone (O_3_) to produce nitrogen dioxide (NO_2_), which is sensitively detected by its chemiluminescence.

A 100 mM NO_3_^−^ stock solution was prepared by dissolving 84.9 mg NaNO_3_ (Sigma Aldrich, Hamburg, Germany) in 10 mL deionized water. Standard dilutions (10 nM, 50 nM, 100 nM, 500nM, 1 µM, 5 µM, 10 µM, and 100 µM NO_3_^−^) were prepared and injected in duplicates to create a calibration curve before the experiment.

All samples were deproteinized prior to analysis using ethanol precipitation. For this, the cell culture supernatants as well as the sera were diluted 1:3 with chilled pure ethanol (0°C). After 30 min precipitation time, the samples were centrifuged at 14000 x *g* for 15 min and the supernatant was used for the experiment.

NO concentrations of the cell culture supernatant as well as of the STEMI and CTR sera were analyzed by injecting 50 μL duplicates of each sample into a purge vessel containing a solution of VCl_3_ (50 mmol/L; Sigma-Aldrich, Hamburg, Germany; catalog: 208272) in hydrochloric acid (HCl; 1 mol/L; Sigma-Aldrich, Hamburg, Germany; catalog: 1098214) at 95°C, continuously purged with a stream of nitrogen gas, connected to the NOA-280i. A gas bubbler between the purge vessel and the NOA-280i was filled with 15 mL of 1 M aqueous NaOH solution (Sigma-Aldrich, Hamburg, Germany; catalog: 655104) to prevent HCl vapors from entering the NOA-280i. Concentrations were calculated using the manufacturer’s NO Analysis Software for Liquid (Version 3.21/Liquid, GE Water & Process Technologies, Analytic Instruments; Boulder, CO, USA).

### 2.4 Cell isolation and culture

Primary endothelial cells (“human umbilical vein endothelial cells”; HUVEC) were isolated as described previously in detail (25) and cultivated in Gibco Medium 199 supplemented with fetal calf serum 10% (FCS; Gibco, Carlsbad, CA, USA), penicillin/streptomycin 1% (100 U/mL, 100 mg/mL; Gibco, Carlsbad, CA, USA), large-vessel endothelial supplement 1% (Gibco, Carlsbad, CA, USA), and heparin (5000 U/mL; Biochrom, Schaffhausen, Switzerland) at 37°C, 21% O_2_ and 5% CO_2_. Umbilical cords were donated by patients giving birth in the Marien-Krankenhaus Luebeck and the University Medical Centre Schleswig-Holstein Campus Luebeck (approved by Local Ethics Committee Cases: 18–325 and 2023–520_1).

EA.hy 926 endothelial cells (kindly provided by Cora-Jean S. Edgell, University of North Carolina, Chapel Hill, NC, USA) were grown in culture as described elsewhere (26). Briefly, cells were grown in 12.5 cm² falcon tissue culture flasks (Corning Inc., Corning, NY, USA; catalog: 353107) until they reached confluence. Cells were grown in Dulbecco’s modified eagle’s medium (DMEM; Thermo Fisher Scientific, Hamburg, Germany; catalog: 41966–029) supplemented with FCS (10%) and penicillin/streptomycin (100 U/mL, 100 mg/mL) at 37°C, with 21% O_2_ and 5% CO_2_.

HUVEC monolayers were stimulated with the complement anaphylatoxin C5a (Merck, Darmstadt, Germany; catalog: 204902). Different concentrations of C5a (0, 1, 10, 50, and 100 ng/mL) as well as different stimulation durations (0, 30, 60, and 120 min and 24 h) were initially tested for their effect on cortical stiffness. In further experiments, HUVEC were stimulated with 50 ng/mL of C5a with or without the C5a-Receptor-1 antagonist (C5aRA) PMX53 (Sigma Aldrich, Hamburg, Germany; catalog number 533683), according to the manufacturer’s instructions [concentration 1 µg/mL (1:1000)] for 24 h. In addition, the endothelial cells were stimulated with 10% patient (STEMI) or healthy donor (CTR) sera (instead of FCS) for 24 h either with or without C5aRA (PMX53). The concentration of C5a in the patient sera was determined by ELISA. The patient group with a concentration of C5a in the lowest quartile is hereinafter referred to as LOW and the group of patients with a concentration in the top quartile as HIGH. Differences in cortical stiffness between HIGH and LOW C5a were determined using AFM measurements.

### 2.5 Atomic force microscopy

The height and stiffness of the eGC and cortical stiffness in both HUVEC and mouse aortic endothelial cells were determined using the AFM-based nanoindentation technique, as described previously (25). Indentation measurements were performed on living confluent cells at 37°C in HEPES-buffered solution (standard composition in mmol/L: 140 NaCl, 5 KCl, 1 MgCl_2_, 1 CaCl_2_, 5 glucose, and 10 HEPES, pH 7.4). To determine cortical stiffness, a Nanoscope Multimode8 AFM (Veeco, Mannheim, Germany) was used. For measuring the nanomechanical properties of the eGC of HUVEC as well as for measurements of ex vivo mouse aortas, a Nanowizard4 (JPK BioAFM Business, Berlin, Germany) was employed.

Briefly, a laser beam was aligned on the back of a gold-coated triangular cantilever (Novascan Technologies, Boone, NC, USA) with a mounted spherical tip (diameter 10 μm) and a nominal spring constant of 10 pN/nm (for eGC) and 30 pN/nm (for cortical stiffness). The cantilever indents the endothelial cell surface with a loading force of 0.5 nN. The reflection of a laser beam is used to quantify the cantilever deflection. The height of the eGC can be calculated by knowing the cantilever force, the piezo displacement, and the deflection sensitivity. For each experimental condition, a total of 150–300 single cells were measured. For each single cell, 6–8 force distance curves were generated and averaged, resulting in n = 900–2400 per condition. Data were collected using the Research NanoScope version 9.20 (64 bit; Bruker Nano GmbH) and calculated using the Protein Unfolding and Nano-Indentation Analysis Software (PUNIAS 3D; Version 1.0; Release 2.3; Copyright 2009).

### 2.6 Fluorescence staining and microscopy

Fluorescence staining and microscopy of the cortical F-actin and of components of the eGC were applied as described preciously (25). Briefly, HUVEC were fixed with either 4% paraformaldehyde or 0.1% glutaraldehyde for further staining. Cortical F-actin was stained using phalloidin-tetramethylrhodamine (10 mg/mL; Sigma Aldrich) after permeabilization of the cells with 0.1% Triton X-100 (Sigma Aldrich; catalog: T8787–50ML) for 10 min. Coverslips were mounted overnight at 4°C with Dako mounting medium (Dako, Carpinteria, CA; catalog: GM30411–2). Wheat germ agglutinin (WGA; conjugate Alexa-Fluor488; Thermo Fisher, Waltham, MA) was used as overview staining for eGC components. After fixation, cells were incubated with 1:500 dilutions of WGA and mounted overnight. For immunostaining of syndecan-1 (CD138), fixed cells were incubated with the primary antibody (1:100, mouse antihuman CD138; monoclonal antibody; Bio-Rad, Hercules, CA; catalog: MCA2459). After incubation, the coverslips were incubated with the secondary antibody (1:400, goat anti-mouse conjugate Alexa-488; Invitrogen, Carlsbad, CA; catalog: A28175) and mounted with Dako mounting medium containing Hoechst solution (1.5 mg/mL; Sigma Aldrich, Hamburg, Germany; catalog: 94403) to stain cell nuclei.

Images were rendered with a Keyence fluorescence microscope BZ9000 (Keyence Corp., Osaka, Japan; magnification x60) using the BZ Viewer/Analyzer-II (software version 2.2; Keyence Corp.). Images and stacks of WGA and phalloidin staining were analyzed for fluorescence intensity (in arbitrary units) using ImageJ software. For analyzing the amount of syndecan-1 per cell relative to control, fluorescence-dot nuclei colocalization was measured using YT Evaluation software (Version 2.1.12014; 64 bit; Synentec, Elmshorn, Germany).

### 2.7 Single-cell force spectroscopy and quantitative monocyte adhesion measurements

Adhesion forces between the endothelial surface and monocytes were quantified by single-cell force spectroscopy and by monocyte wash-away assays as described elsewhere (27). Human monocytes were isolated from the blood of healthy donors using the S-pluriBead Maxi Reagent Kit (pluriSelect Life Science, Leipzig, Germany; catalog number 70–50010-12) following the manufacturer’s instructions.

For single-cell force spectroscopy a single human monocyte was attached to the AFM cantilever in order to measure the adhesion forces between the monocyte and HUVEC monolayers. Measurements were performed by using the Nanowizard4 CellHesion-Module (JPK BioAFM Business, Berlin, Germany). Arrow TL-2 tipless cantilevers (NanoAndMore GmbH, Wetzlar, Germany) were incubated prior to all experiments for 20 min in Corning Cell-Tak (Fisher Scientific GmbH, Schwerte, Germany; catalog: 10317081) to attach the monocyte to the cantilever. For quantifying the adhesion forces between the monocyte and the endothelial cells, the monocyte was brought into contact with the endothelial surface for 10 s with a constant set point of 0.5 V and then pulled away to obtain force-distance/adhesion curves. The maximal adhesion forces (in N) between monocyte and endothelial surface and the adhesion energy (in J) were measured and analyzed using the JPK Data processing software version 7.0.112 (Bruker Nano GmbH, Berlin, Germany).

In addition, monocyte-endothelium interaction was quantified by monocyte wash-away assays. For this, monocytes were fluorescently labeled using an Alexa Flour 488 anti-human CD14 antibody (25:1000, Biolegend, San Diego, CA, USA; catalog: 367130) and added to a confluent HUVEC monolayer for 4 h. To remove nonadherent monocytes, cells were washed carefully four times with PBS, following a standardized protocol. HUVEC and adherent monocytes were fixed with 4% paraformaldehyde and subjected to fluorescence microscopy for further analysis.

### 2.8 Preparation of mouse aortas/assessment of mouse aortic endothelial cell nanomechanics

Wild-type (B6.JRj), C5ar1^–/–^ (B6.129S4-C5ar1^tm1Cge^), C5^-/-^ (B6(Cg)Tg(Ins2-GP)zbz) and mice double deficient for C5aR1 and CXCL4 (C5aR1^-/^-CXCL4-^/-^) (on C57BL/6J background) were bred and maintained in an SPF animal facility of the University of Luebeck (Case: 39.2_2020–08-20_Karsten) as described previously (28). All study animals were between 8 and 18 weeks of age and handled in accordance with the appropriate institutional and national guidelines. Both male and female mice were equally used for the experiments.

The aortas were harvested and prepared for ex vivo analysis by AFM, as described previously (29), to assess the thickness and stiffness of the eGC and cellular cortex of the mouse aorta endothelial cells using the AFM.

Briefly, aortas were harvested after sacrificing the animals and freed from the surrounding tissue. Small patches (~1mm²) of the aorta were attached on Cell-Tak-coated glass coverslips with the endothelial cells facing upwards, making them accessible for further experiments. After preparation, the ex vivo patches were cultured in minimal essential medium (Invitrogen Corp., La Jolla, CA, USA) supplemented with 10% FCS (Gibco, Carlsbad, CA, USA), 1% minimal essential medium vitamins (Invitrogen), 1% minimal essential medium nonessential amino acids (Invitrogen), and 1% penicillin/streptomycin (100 U/mL, 100 mg/mL). Integrity of the ex vivo endothelial cell monolayers derived from mouse aorta was confirmed by immunostaining of platelet endothelial cell adhesion molecule 1 (PECAM1; data not shown), and individual cells on those preparations were studied.

### 2.9 Small G-protein activation assays

The intracellular concentrations of GTP-bound Rac1 and RhoA GTPases were measured by using colorimetric G-LISA activity measurements (G-protein ELISA assays; Cytoskeleton, Denver, CO, USA.; catalog: Rac1: BK128; RhoA: BK124). HUVEC were serum starved for 24 h and treated either with C5a or STEMI vs. CTR serum +/- C5aR1 antagonist. After processing, cell lysates were subjected to the G-LISA according to the manufacturer’s instructions. The final reaction absorbance was measured using a Mitras LB940 microplate reader (Berthold Technologies, Bad Wildbad, Germany). Absorbance was detected at 490 nm. Presented data are background subtracted.

### 2.10 RNA extraction and quantitative real−time PCR

RNA from primary HUVEC was extracted using the innuPREP RNA Mini Kit 2.0 (IST Innu-screen GmbH, Berlin, Germany; catalog: 12183020) according to the manufacturer’s protocol. cDNA of total RNA (1000 ng) was synthesized with the Go Script Reverse Transcriptase (Promega GmbH, Walldorf, Germany; catalog: A5003) and random hexamer primers (Thermo Fisher Scientific; catalog: SO142) according to the instructions of the manufacturer.

Sequences: C5aR1 (Forward: 5´-GGCAGTGGTGGCCAGTTTCT-3´; Reverse: 5´-GGGAGGCATTTCCGCAGTGC-3´), L28 (Forward: 5′-ATGGTCGTGCGGAACTGCT-3′; Reverse: 5′-TTGTAGCGGAAGGAATTGCG-3′).

L28 primers were synthesized by Invitrogen (Invitrogen, Carlsbad, CA); C5aR1 primers were synthesized by Metabion (Metabion International AG, Planegg, Germany). Quantitative real-time polymerase chain reaction (RT-PCR) was performed in the Eco48 qPCR System (PCRmax Limited Beacon Road, Staffordshire, United Kingdom) using 1 μg cDNA and the SensiMix SYBR Kit (Bioline, Luckenwalde, Germany; catalog: QT615–05) in a total volume of 12.5 μL per assay.

The cutoff point (Ct) was defined as the value when the fluorescent signal increased above the background threshold. Gene-specific mRNA expression of C5aR1 was normalized to mRNA expression of the housekeeping gene ribosomal protein L28. Relative expression values were calculated using the 2^-(ΔΔCT) method and are presented as the relative fold change.

### 2.11 Wound healing assay

For wound closure experiments, EA.hy926 endothelial cell monolayers were scratched with a 200-μL sterile pipette tip and detached cells were washed away with PBS. Stimulation with C5a or STEMI vs. CTR serum +/- C5aR1 antagonist was carried out in HEPES-buffered solution. Once the scratch was made, the culture flasks were transferred into a flask heater set to 37°C.

Wound closure was analyzed with time-lapse video microscopy using an Olympus CKX53 microscope (EVIDENT Europe GmbH, Hamburg, Germany) with a 10x objective. Pictures were taken every 5 min for 24 h with a VWR VisiCam 5 (VWR International GmbH, Darmstadt, Germany) using the OPTIKA Vision Software (Version 2.13; OPTIKA S.r.l., Ponteranica, Italy). To quantify the cell migration characteristics (wound closure in %), the images were analyzed using an ImageJ (ImageJ software version 1.52a; NIH, Bethesda, MD; https://imagej.nih.gov/ij/download.html, last accessed 8 Feb 2024) plugin for the high-throughput image analysis of *in vitro* scratch wound healing assays developed by Suarez-Arnedo et al (30). The wound closure speed was furthermore normalized into the cell growth rate (µm/h) to render all data comparable, as the cell front velocity is independent of the initial gap width, gap creation method, gap orientation, the microscopy settings, and the objective lens` magnification. For this, the wound closure speed (surface area per time) was determined by creating a graph showing the cell-covered area per time and defining the linear part of the curve (trend line calculation). The slope of the trend line corresponds to the growth rate. To finally calculate the cell front velocity, the wound closure speed was divided by the length of the cell front (in µm). (For further information compare the ibidi Application Note 30 on Experimental Setup Optimization and Data Analysis of Wound Healing Assays; ibidi GmbH, Gräfelfing, Germany; https://www.ibidi.com/img/cms/support/AN/AN30_Wound_Healing_Data_Analysis.pdf, last accessed Feb. 09th 2023).

### 2.12 Sprouting angiogenesis aortic ring assay

The sprouting angiogenesis aortic ring assay was performed as described, previously (31), with some modifications: Aortas of mice were harvested as described above and sectioned into 1 mm pieces. Matrigel (Corning) was selected for its basement membrane-like composition as embedding medium. 200 μL of Matrigel was evenly coated on 12-well plates placed on ice and polymerized by incubating in a humidified incubator at 37°C with 5% CO_2_ for 30 min. Subsequently, freshly obtained aortic rings were placed at the center of each well, and an additional 300 μL of Matrigel was applied on top of the aortic ring tissue. The plates were re-incubated under the same conditions until the Matrigel was fully polymerized. The Matrigel was supplemented in its liquid state with VEGF-A (25 ng/mL, Sigma Aldrich). Following Matrigel polymerization, the aortic ring sandwich assay was incubated in 1000 μL of endothelial culture medium supplemented with 2% FCS and 1% penicillin/streptomycin. Media were replenished every 2 days throughout the assay.

To monitor sprouting in real-time, the explants were placed under a light microscope equipped with phase-contrast optics and a digital camera with a Visiscope inverted microscope (IT404, VWR, Radnor, PA, USA). Sprouting was typically observed by day 3, with maximal sprouting occurring around day 7. Sprouting was quantified using the Angiogenesis analyzer for ImageJ as described before (32).

### 2.13 Tube formation assay

The tube formation assay was performed, as described, previously (33). For the *in vitro* tube formation assay, MHEC-5T (6 x 10^4^ cells/well) were plated onto Matrigel-coated Angiogenesis µ-slides (ibidi, Planegg, Germany) in endothelial culture medium containing 2% FCS. Cells were co-incubated with C5a (R&D) at different concentrations. After 6h tube formation was imaged by phase-contrast microscopy. Tube Formation was analyzed using Angiogenesis analyzer for ImageJ as described before.

## 3 Results

### 3.1 C5a concentrations and eGC components are increased after myocardial infarction and alter endothelial function in STEMI patients

For forming the experimental groups, C5a concentrations were measured using ELISA in the STEMI patients’ serum. The sera were further sorted by quartile according to their level of C5a concentration. The lowest quartile as well as the top quartile were pooled to form the experimental groups LOW (mean C5a concentration: 20.43 ± 2.8 ng/mL) and HIGH (mean C5a concentration: 88.56 ± 6.9 ng/mL). Serum of healthy donors was implemented as control group (CTR; mean C5a concentration: 3.61 ± 1.1 ng/mL).

**Figure 1:**
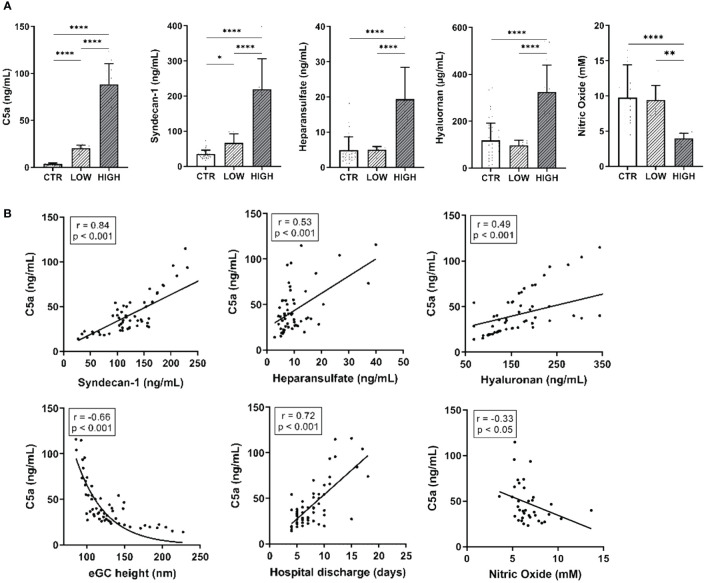
C5a Concentrations and eGC Components Are Increased After Myocardial Infarction and Alter Endothelial Function in STEMI Patients. **(A)** Serum levels of C5a, syndecan-1, heparan sulfate, and hyaluronan (measured by enzyme-linked immunosorbent assay, ELISA) as well as nitric oxide (NO) concentrations (measured chemiluminescence based). **(B)** Correlation of C5a concentrations in ST-elevation myocardial infarction (STEMI) patient sera vs. syndecan-1, heparan sulfate, and hyaluronan as well as of endothelial glycocalyx (eGC) height, days until discharge from hospital, and NO concentration (calculated without data from LOW group). Rho (r) and p-values (p) shown for correlations. Groups: LOW: STEMI patient sera of the lowest C5a concentration quartile; HIGH: STEMI patient sera of the top C5a-concentration quartile; CTR: healthy donors. p-values: ****p<0.0001; **p<0.01; *p<0.05.

Concentrations of both C5a and the eGC components syndecan-1, heparan sulfate, and hyaluronan were significantly increased in the HIGH group compared to controls (all: p<0.0001) (**Figure 1A**). NO bioavailability was decreased by 52% in the HIGH group compared to CTR (p<0.0001).

The eGC components measured in the patients’ blood correlated with the C5a concentration (**Figure 1B**). The C5a concentration positively correlated with syndecan-1 (r=0.84; p<0.001), heparan sulfate (r=0.53; p< 0.001) and hyaluronan (r=0.49; p<0.001). NO concentration (r=-0.33; p<0.05; calculated without LOW data) as well as eGC height (r=-0.66; p<0.001) negatively correlated with C5a. Of note, C5a concentrations correlated positively with the days until hospital discharge (r=0.72; p<0.001).

### 3.2 C5a incrementally changes the nanomechanical properties of the endothelial surface layers in a concentration-dependent manner

To quantify nanomechanic changes of the eGC and cellular cortex confluent HUVEC monolayers were treated with 10% patient or control sera and the nanomechanical properties (height and stiffness) of the endothelial surface layers (eGC and cortex) were probed by using the AFM nanoindentation technique.

**Figure 2:**
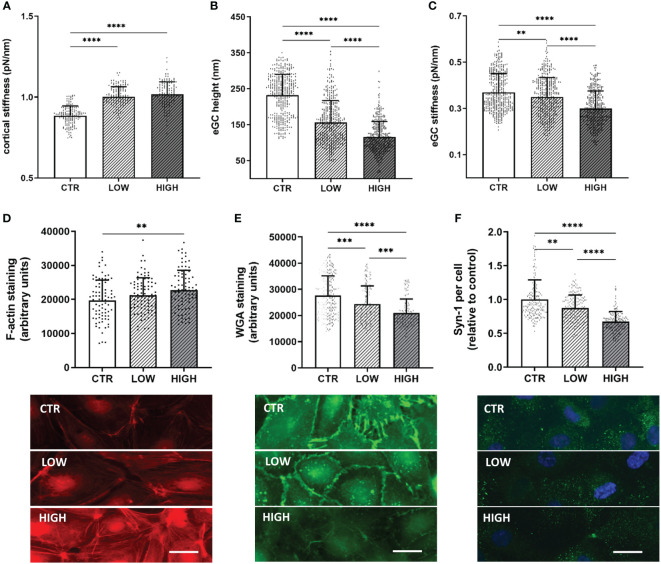
C5a Incrementally Changes the Nanomechanical Properties of the Endothelial Surface Layers in a Concentration-Dependent Manner **(A–C)** Statistical analysis of atomic force microscopy (AFM) nanoindentation measurements of human umbilical vein endothelial cells (HUVEC) monolayers. Data showing mean ± SD of **(A)** cortical stiffness, **(B)** endothelial glycocalyx (eGC) height and **(C)** eGC stiffness. Each dot represents a single cell measurement (8 force-distance curves per dot; N=6). **(D–F)** Representative fluorescence images and statistical fluorescence intensity analyses. **(D)** Phalloidin-tetramethylrhodamine–stained (red: F-actin) and **(E)** Wheat germ agglutinin (WGA)-stained (green; representing unspecific eGC components) HUVEC monolayers. Graphs showing the measured fluorescence intensity in arbitrary units. **(F)** Syndecan-1 antibody-stained HUVEC monolayers (green: syndecan-1; blue: cell nucleus) after stimulation. Graphs showing the amount of syndecan-1 per cell relative to the control group. (**D–F**: N=5; scale bars: 50 µm). Groups: LOW: ST-elevation myocardial infarction (STEMI) patient sera of the lowest C5a concentration quartile; HIGH: STEMI patient sera of the top C5a -concentration quartile; CTR: healthy donors. p-values: ****p<0.0001; ***p<0.001; **p<0.01.

Incubation with LOW serum increased the cortical stiffness by 10% compared to control-treated HUVEC (CTR vs. LOW: 0.88 ± 0.06 pN/nm vs. 1.0 ± 0.06 pN/nm; p<0.0001) (**Figure 2A**). Treatment with HIGH serum augmented this effect, increasing the cortical stiffness further by 12% (p<0.0001) compared to healthy controls.

eGC height was decreased by 32% (p<0.0001) after treatment with LOW serum and by 49% after HIGH serum C5a concentrations compared to controls (CTR vs. HIGH: 232.4 ± 57.7 nm vs. 115.3 ± 43.4 nm; p<0.0001) (**Figure 2B**). After treatment with HIGH serum, eGC damage was markedly stronger than after treatment with LOW serum (LOW overall mean 156.3 ± 60.1 nm vs. HIGH overall mean 115.3 ± 43.4 nm; p<0.0001). In addition, the eGC stiffness was impaired after serum stimulation with a decreased stiffness of 6% in the LOW (p<0.01) and 19% in the HIGH group (CTR vs. HIGH: 0.37 ± 0.08 pN/nm vs. 0.29 ± 0.07 pN/nm; p<0.0001) (**Figure 2C**). This combination of loss of height and reduction in stiffness of the eGC indicates shedding of the glycocalyx after stimulation with STEMI serum (20).

Results of fluorescence staining were consistent with the AFM findings. Fluorescence staining of the cortical F-actin conformed with the results of the AFM-based cortex measurements. Fluorescence intensity was increased in the LOW (7%) and the HIGH (15%; p<0.01) group compared to CTR (**Figure 2D**), indicating polymerization of cortical actin. eGC shedding detected by AFM could be confirmed by fluorescence staining of eGC components, as STEMI-induced eGC deterioration resulted in a reduced WGA fluorescence intensity in LOW (12%; p<0.001) and HIGH (24%; p<0.0001) serum-treated cells compared to the CTR group (**Figure 2E**). Additionally, the specific syndecan-1-antibody (anti-CD138) showed a reduction in syndecan-1 per cell after serum stimulation compared to CTR. Both groups, LOW (by 13%; p<0.01) and HIGH (by 33%; p<0.0001), showed significant reductions in syndecan-1 per cell compared to the control-treated HUVEC (**Figure 2F**).

### 3.3 C5a stimulation leads to cortical stiffening and eGC degradation

To evaluate the contribution of C5a on endothelial nanomechanics and condition of the eGC, different concentrations of C5a (0, 1, 10, 50, and 100 ng/mL) and different stimulation durations (0, 30, 60, and 120 min and 24 h) were applied to HUVEC monolayers. The nanomechanical properties (height and stiffness) of the cellular cortex and the eGC were quantified via AFM nanoindentation technique.

**Figure 3:**
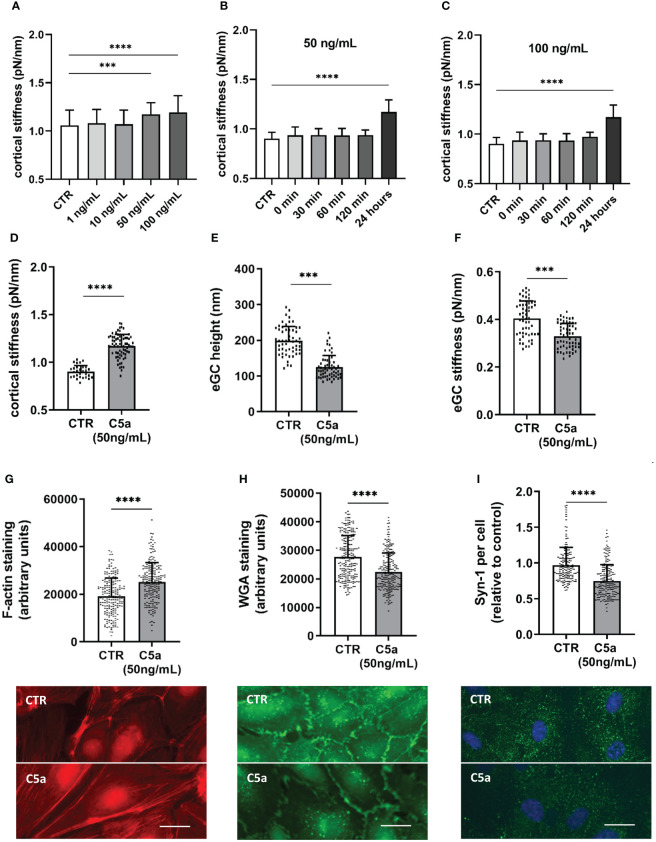
C5a stimulation leads to cortical stiffening and eGC degradation. Different concentrations of C5a as well as different stimulation durations were tested for their influence on cortical stiffness measured via atomic force microscopy (AFM) nanoindentation technique. **(A)** Statistical analysis of AFM measurements of human umbilical vein endothelial cells (HUVEC) monolayers. Data showing mean ± SD of cortical stiffness after stimulation with different concentrations of C5a (0, 1, 10, 50 und 100 ng/mL; N=5). **(B)** Statistical analysis of AFM measurements of HUVEC monolayers showing mean ± SD of cortical stiffness after stimulation with 50 ng/mL of C5a using different stimulation durations (0, 30, 60, and 120 min and 24 h; N=5). **(C)** Statistical analysis of AFM measurements of HUVEC monolayers showing mean ± SD of cortical stiffness after stimulation with 100 ng/mL of C5a using different stimulation durations (0, 30, 60, and 120 min and 24 h; N=5). **(D–F)** Statistical analysis of AFM nanoindentation measurements. Data showing mean ± SD of **(D)** cortical stiffness, **(E)** eGC height, and **(F)** eGC stiffness after stimulating HUVEC monolayers with 50 ng/mL of C5a for 24 h (N=5). **(G–I)** Representative fluorescence images and statistical fluorescence intensity analyses. **(G)** Phalloidin-tetramethylrhodamine-stained (red: F-actin) and **(H)** Wheat germ agglutinin (WGA)-stained (green; representing unspecific eGC components) HUVEC monolayers after stimulation as described. Graphs showing the measured fluorescence intensity in arbitrary units. **(I)** Syndecan-1 Antibody-stained HUVEC monolayers (green: syndecan-1; blue: cell nucleus) after stimulation. Graphs showing the amount of syndecan-1 per cell relative to the control group. (**G–I**: N=6; scale bars: 50 µm). p-values: ****p<0.0001; ***p<0.001.

Stimulation with 50 ng/mL (p<0.001) and 100 ng/mL (p<0.0001) induced significant stiffening of the endothelial cortex (**Figure 3A**). Both concentrations were further tested in a temporal context and showed a significant increase in cortical stiffness after 24 h of stimulation (**Figures 3B, C**, both p<0.0001). In subsequent experiments, 50 ng/mL (hereinafter referred to as C5a group) was applied, as this concentration proved to be the lowest with a significant effect on nanomechanical properties of the vascular surface.

Cortical stiffness increased by 29% after C5a stimulation compared to control conditions (CTR vs. C5a: 0.9 ± 0.06 pN/nm vs. 1.17 ± 0.12 pN/nm; p<0.0001) (**Figure 3D**). Compared to control treatment, eGC height was diminished by 36% (CTR vs. C5a: 198.5 ± 40.0 nm vs. 124.5 ± 32.7 nm; p<0.001; **Figure 2E**) and the eGC stiffness was reduced by 19% (CTR vs. C5a: 0.40 ± 0.07 pN/nm vs. 0.32 ± 0.05 pN/nm; p<0.001; **Figure 3F**) after stimulation with 50 ng/mL of C5a. Both effects (**Figures 3E, F**) were observed after treatment with C5a, indicating eGC shedding.

The nanoindentation measurements were again confirmed by fluorescence staining of the cellular cortex and the eGC. The fluorescence signal for cortical F-actin was enhanced by 31% (p<0.0001), indicating polymerization of cortical actin (**Figure 3G**). Unspecific staining of the eGC using WGA revealed a reduced fluorescence intensity of the C5a-treated cell surface by 19% (p<0.001) (**Figure 3H**), which was additionally underpinned by the syndecan-1 staining showing a reduction in syndecan-1 per cell by 26% compared to controls (p<0.001) (**Figure 3I**).

### 3.4 C5a-receptor-1 antagonist (PMX53) reduces impairment of the vascular surface after AMI and C5a stimulation

After observing shedding of the eGC and cortical stiffening after treatment with both patient sera and recombinant C5a, C5aRA (PMX53) was employed to investigate the influence of the C5a:C5aR1 axis on vascular surface nanomechanics and eGC integrity. To test the effect of C5aRA, two different experimental approaches were followed: i) stimulation with CTR vs. C5a (50 ng/mL) with and without C5aRA treatment; and ii) stimulation with STEMI patient serum (STEMI group: mean C5a concentration: 86.74 ± 6.5 ng/mL) vs. healthy control serum (CTR group: mean C5a concentration: 3.61 ± 1.1 ng/mL) with and without C5aRA treatment. The mean C5a concentration of the STEMI group corresponds to the mean C5a concentration of the HIGH group. The nanomechanical properties of the cellular cortex and the eGC were probed using the AFM nanoindentation technique on HUVEC monolayers.

**Figure 4:**
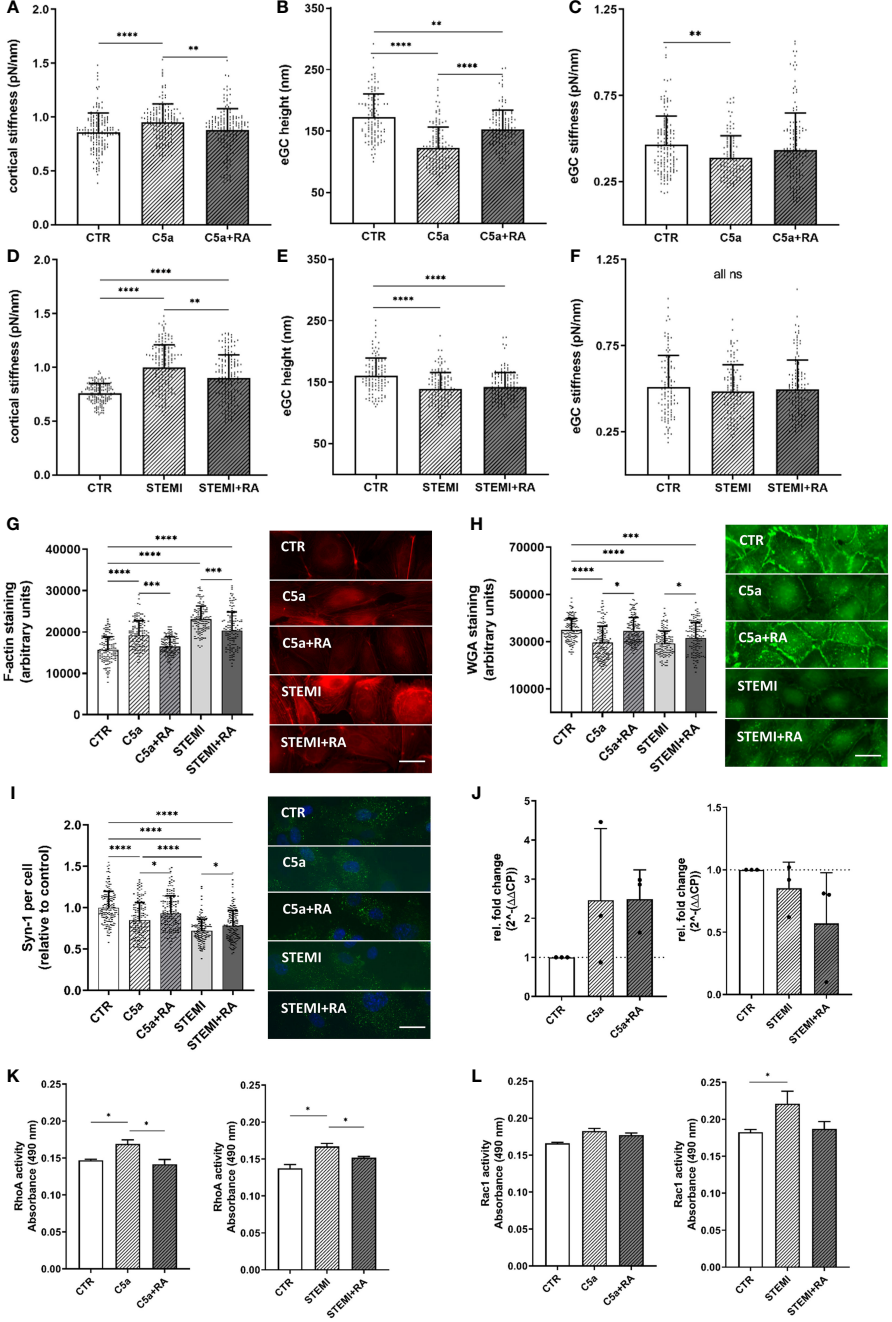
C5a-receptor-1 antagonist (PMX53) reduces impairment of the vascular surface after AMI and C5a stimulation. **(A–C)** Statistical analysis of atomic force microscopy (AFM) nanoindentation measurements of human umbilical vein endothelial cells (HUVEC) monolayers. Data showing mean ± SD of **(A)** cortical stiffness, **(B)** endothelial glycocalyx (eGC) height, and **(C)** eGC stiffness of CTR, C5a, and C5a+RA groups. Each dot represents a single cell measurement (8 force-distance curves per dot; N=5) as described in detail in the methods section. **(D–F)** Statistical analysis of AFM nanoindentation measurements of HUVEC monolayers. Data showing mean ± SD of **(D)** cortical stiffness, **(E)** eGC height, and **(F)** eGC stiffness of CTR, STEMI, and STEMI+RA groups. Each dot represents a single cell measurement (8 force-distance curves per dot; N=5) as described in detail in the methods section. **(G–I)** Representative fluorescence images and statistical fluorescence intensity analyses. **(G)** Phalloidin-tetramethylrhodamine-stained (red: F-actin) and **(H)** Wheat germ agglutinin (WGA)-stained (green; representing unspecific eGC components) HUVEC monolayers after stimulation as described. Graphs showing the measured fluorescence intensity in arbitrary units. **(I)** Syndecan-1 antibody-stained HUVEC monolayers (green: syndecan-1; blue: cell nucleus) after stimulation. Graphs showing the amount of syndecan-1 per cell relative to the control group. (**G–I**: N=5; scale bars: 50 µm). **(J)** Expression levels of C5a-Receptor1 genes via quantitative PCR after stimulation of HUVEC. Gene-specific mRNA expression was measured using the ΔΔCt method relative to expression of ribosomal protein L28 (endogenous control) and normalized to unstimulated controls (N=3). **(K)** Small GTPases activation analysis of RhoA from confluent HUVEC. Bar graphs show raw optical density (O.D.) measured with an absorbance wavelength of 490 nm (N=3). **(L)** Small GTPases activation analysis of Rac1 from confluent HUVEC after treatment. Bar graphs show raw optical density (O.D.) measured with an absorbance wavelength of 490 nm (N=3). Groups: CTR: stimulation with standard cell culture media; C5a: cell culture media + 50 ng/mL of C5a; C5a+RA: cell culture media + 50 ng/mL of C5a + C5a-Receptor antagonist (PMX53; 1:1000). Serum groups: CTR: stimulation with cell culture media + 10% Serum of healthy donors; STEMI: cell culture media + 10% serum of STEMI patients (final C5a concentration 8.7 ng/mL); STEMI+RA: cell culture media + 10% serum of STEMI patients + C5a-Receptor antagonist (PMX53; 1:1000). p-values: ****p<0.0001; ***p<0.001; **p<0.01; *p<0.05.

Nanoindentation of the cellular cortex revealed increased stiffness after C5a stimulation (CTR vs. C5a: 0.85 ± 0.18 pN/nm; vs. 0.95 ± 0.17 pN/nm; p<0.0001). However, treatment with C5aRA fully restored the cortex stiffness compared to CTR levels (**Figure 4A**). After C5a stimulation the condition of the eGC changed and height was decreased (CTR vs C5a: 172 ± 37 nm vs. 123 ± 34 nm; p<0.0001) compared to controls (**Figure 4B**). Although treatment with C5aRA did not completely regenerate the eGC height up to control levels, there was a significant improvement in the C5a+RA group compared to the C5a group (C5a vs. C5a+RA: 122.7 ± 33.8 nm vs. 152.9 ± 30.8 nm; p<0.0001). C5a-mediated shedding of the eGC led to softening of the eGC by 17% compared with CTR (p<0.01) (**Figure 4C**) and treatment with C5aRA to full restoration of the eGC stiffness compared to controls, both indicating recovery of the eGC.

Treatment with C5aRA could reduce the STEMI-induced cortical stiffening (STEMI vs. STEMI+RA: 0.99 ± 0.2 pN/nm vs. 0.9 ± 0.2 pN/nm; p<0.01) (**Figure 4D**). STEMI serum reduced eGC height significantly compared to controls (CTR vs. STEMI: 161 ± 29 nm vs. 138 ± 26 nm; p<0.0001) (**Figure 4E**). This effect could not be prevented by C5aRA treatment. eGC stiffness was not affected after stimulation with the C5aR inhibitor (**Figure 4F**).

Treatment with C5aRA prevented the C5a-induced polymerization of cortical actin, confirming the results of AFM-based nanoindentation measurements. Simultaneously, C5aRA treatment in the context of STEMI reduced cortical F-actin (STEMI vs. STEMI+RA: p<0.001) (**Figure 4G**).

The AFM-based detection of eGC shedding and cortical stiffening were also visualized by fluorescence staining. WGA staining revealed a reduction in eGC components after both C5a (p<0.0001) and stimulation with STEMI serum (p<0.0001) (**Figure 4H**).

Treatment with C5aRA fully restored the eGC components, reaching control levels after C5a stimulation, and the condition of the eGC was significantly improved after stimulation with STEMI serum (STEMI vs. STEMI+RA: p<0.05) (**Figure 4H**). Consistently, syndecan-1 staining indicated a reduction in syndecan-1 per cell both after stimulation with C5a (p<0.0001) and with STEMI serum (p<0.0001) compared with healthy controls (**Figure 3I**). Fullly restored syndecan-1 levels were detected after C5aRA treatment (C5a vs. C5aRA: 0.85 ± 0.2 vs. 0.93 ± 0.2 Syn-1 per cell (relative to control); p<0.05) (**Figure 4I**). STEMI+RA stimulation resulted in a significant elevation in stainable syndecan-1 per cell (STEMI vs. STEMI+RA: 0.71 ± 0.1 vs. 0.78 ± 0.2 Syn-1 per cell (relative to control); p<0.05) (**Figure 4I**).

Induction of C5a-Receptor-1 expression was confirmed by measuring C5a-Receptor-1 genes via quantitative PCR after stimulating HUVEC with C5a but not with STEMI serum (**Figure 4J**). To determine the pathway of cellular cortex stiffening, small GTPases were quantified. For this, RhoA and Rac1 were measured as key mechanotransduction players, regulating actin cytoskeletal tension using G-LISA assays. Analysis of baseline RhoA activity in confluent HUVEC monolayers revealed significantly higher RhoA activity after both C5a and stimulation with STEMI serum (both p<0.05) (**Figure 4K**). RhoA activity was regulated to control levels in both groups after C5aRA treatment. Baseline Rac1 activity was elevated after stimulation with STEMI serum (p<0.05) and reversed by C5aRA treatment (**Figure 4L**).

### 3.5 The influence of C5a and STEMI on eGC condition and cortical stiffness are attenuated in a mouse model for C5a-receptor-1-knockout

To further examine the effects of the C5a-Receptor-1 on vascular surface nanomechanics and eGC integrity ex vivo, endothelial cells derived from C5a-Receptor-1 knock-out (KO; C5aR1^–/–^) mouse aorta were employed.

**Figure 5:**
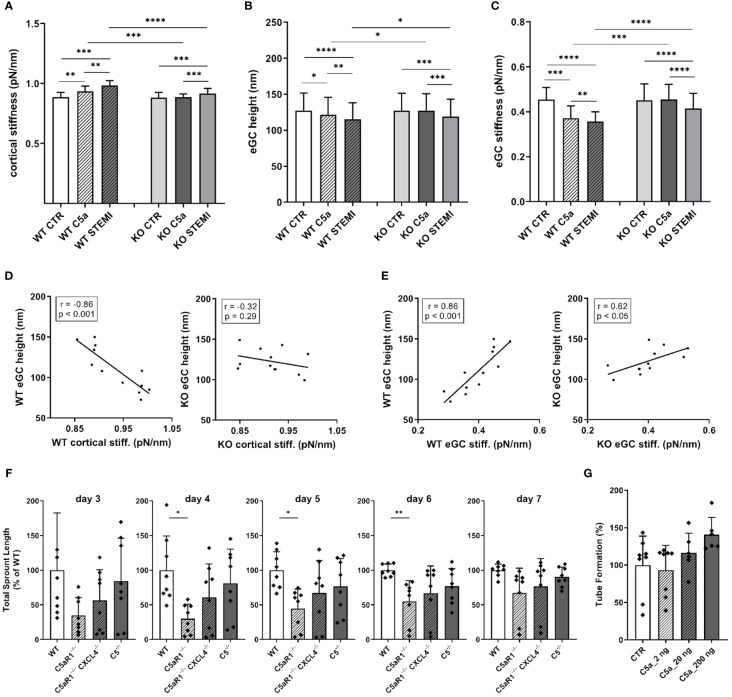
The influence of C5a and STEMI on eGC condition and cortical stiffness are attenuated in a mouse model for C5a-receptor-1-knockout. **(A–C)** Statistical analysis of atomic force microscopy (AFM) nanoindentation measurements of living endothelial cells on isolated mouse aorta preparations. Aortas of wild-type (WT; N=6) and C5a-Receptor-1-knock-out (KO; C5aR1^–/–^; N=6) mice were harvested and made available for AFM measurements as described in detail in the methods section. Groups: Preparations of WT and KO aortas were stimulated with either cell culture media (control group, CTR), cell culture media + 50 ng/mL of C5a (C5a group), or cell culture media + 10% STEMI serum (STEMI group). Data showing mean ± SD of **(A)** cortical stiffness, **(B)** eGC height, and **(C)** eGC stiffness of single cell measurements of living endothelial cells on isolated mouse aorta preparations (8 force-distance curves per single cell; n=50 cells per mouse; N=6 mice per group). **(D)** Correlation of WT endothelial glycocalyx (eGC) height vs. WT cortical stiffness and KO eGC height vs. KO cortical stiffness (CTR and stimulation with STEMI serum). **(E)** Correlation of WT eGC height vs. WT eGC stiffness and KO eGC height vs. KO eGC stiffness (CTR and stimulation with STEMI serum). Rho (r) and p-values (p) shown for correlations. **(F)** Sprouting angiogenesis aortic ring assay of C5^-/-^ (B6(Cg)Tg(Ins2-GP)zbz) and mice double deficient for C5aR1 and CXCL4 (C5aR1^-/^-CXCL4-^/-^) (on days 3, 4, 5, 6 and 7). **(G)** Tube formation assay of MHEC-5T cells after C5a stimulation. p-values: ****p<0.0001; ***p<0.001; **p<0.01; *p<0.05; ns, not significant.

Stimulation with either C5a (p<0.01) or STEMI serum (p<0.001) enhanced cortical stiffness in WT mice (**Figure 5A**). As expected, stimulation with C5a showed no effects on the C5aR1^–/–^ mouse aortic endothelial cells, although stimulation with STEMI serum did cause cortical stiffening compared to both controls and to the C5a group (KO CTR vs. C5a vs. STEMI: 0.88 ± 0.0.4 pN/nm vs. 0.88 ± 0.03 pN/nm vs. 0.92 ± 0.04 pN/nm; p<0.001). Cortical stiffness differed between WT and KO after simultaneous stimulations: after C5a stimulation cortical stiffness was significantly higher in WT mice than in C5aR1^–/–^ mice (WT C5a vs. KO C5a: 0.94 ± 0.04 pN/nm vs. 0.88 ± 0.03 pN/nm; p<0.001). C5aR1^–/–^ mice also showed less cortical stiffening after stimulation with STEMI serum compared to WT mice (WT STEMI vs. KO STEMI: 0.98 ± 0.04 pN/nm vs. 0.92 ± 0.04 pN/nm; p<0.0001) (**Figure 5A**).

Stimulation with C5a (p<0.05) as well as with STEMI serum (p<0.001) reduced eGC height in WT mice (**Figure 5B**). C5a stimulation had no impact on the eGC of C5aR1^–/–^ mice. However, stimulation with STEMI serum reduced eGC height in KO mice by 9% compared to controls (p<0.001). In direct comparison between WT and KO, eGC height was preserved in C5aR1^–/–^ mice after stimulation with C5a (WT C5a vs. KO C5a: 121.5 ± 24 nm vs. 127.2 ± 24 nm; p<0.05) as well as with STEMI serum (WT STEMI vs. KO STEMI: 115.1 ± 23 nm vs. 127.0 ± 23 nm; p<0.05) (**Figure 5B**).

Both C5a (p<0.001) and STEMI serum (p<0.0001) decreased eGC stiffness in WT mice (**Figure 5C**). C5a stimulation had no impact on eGC stiffness in C5aR1^–/–^ mice. stimulation with STEMI serum on KO aortas did decrease eGC stiffness (p<0.001). Compared to C5aR1^–/–^ mice stimulation both with C5a (WT C5a vs KO C5a: 0.37 ± 0.05 vs. 0.46 ± 0.06 pN/nm; p<0.001) and STEMI serum (WT STEMI vs. KO STEMI: 0.36 ± 0.04 vs. 0.42 ± 0.06 pN/nm; p<0.0001) showed a higher impact on eGC stiffness in WT mice (**Figure 5C**).

AFM parameters were correlated within the experimental groups. eGC height was negatively associated with cortical stiffness for WT mice (r=-0.86; p<0.001). This effect was diminished in C5aR1^–/–^ mice, where no significant relationship was observed between eGC height and cortical stiffness (**Figure 5D**). Furthermore, eGC height was positively associated with eGC stiffness in WT mice (r=0.86; p<0.001). This correlation was much weaker in C5aR1^–/–^ mice (r=0.62; p<0.05) (**Figure 5E**).

In order to assess the impact of C5aR1 depletion on the potential of vessel growth, we employed the sprouting angiogenesis aortic ring assay (31). We harvested aorta sections from mice of different genotypes and found that C5aR1^-/-^ mice display reduced sprouting angiogenesis after 4, 5 and 6 days of culture compared to global C5^-/-^ and WT. Previously, we have described a C5aR1-CXCL4 axis in revascularization (34). Interestingly, C5aR1^-/-^ CXCL4^-/-^ double deficient mice did not display this phenotype of reduced angiogenesis (**Figure 5F**). Thus, the C5aR1-CXCL4 axis does not seem to be the driver of the observed effect. When endothelial cells (MHEC-5T) were stimulated with C5a, on the other hand, angiogenic potential measured in the tube formation assay increased at a concentration of 200 ng/ml C5a (**Figure 5G**).

### 3.6 Endothelial function is improved after treatment with C5aRA

**Figure 6:**
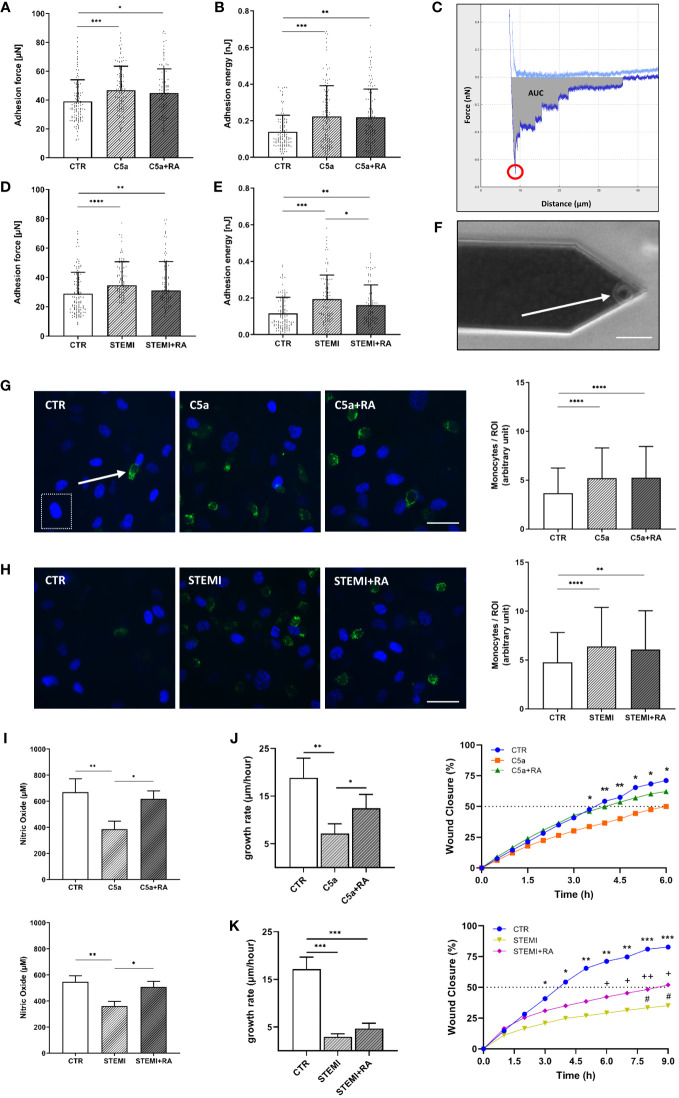
Endothelial function is improved after treatment with C5aRA. **(A)** Data showing mean ± SD of monocyte adhesion forces to human umbilical vein endothelial cells (HUVEC) monolayers measured via atomic force microscopy (AFM)-based single-cell force spectroscopy (SCFS) of control (CTR), C5a, and C5a+RA groups (N=5). **(B)** Mean ± SD of adhesion energy between monocyte and HUVEC monolayer (energy needed to separate monocyte from HUVEC) of CTR, C5a, and C5a+RA groups (N=5) measured via AFM-based SCFS. **(C)** Exemplary force-distance curve of SCFS. Red circle indicates the maximum adhesion force between monocyte and HUVEC monolayer. Gray area representing the total adhesion energy calculated as the area under the curve (AUC). **(D)** Data showing mean ± SD of monocyte adhesion forces to HUVEC monolayers measured via AFM-based SCFS of CTR, ST-elevation myocardial infarction (STEMI), and STEMI+RA groups (N=5). **(E)** Mean ± SD of adhesion energy between monocyte and HUVEC monolayer (energy needed to separate monocyte from HUVEC) of CTR, STEMI, and STEMI+RA groups (N=5) measured via AFM-based SCFS. **(F)** Exemplary picture of AFM cantilever with mounted monocyte (white arrow) before adhesion measurements (scale bar: 25 µm). **(G)** Representative fluorescence images of human monocytes (green: CD14-labeled monocytes; indicated by white arrow) adhesive to HUVEC monolayer of CTR, C5a, and C5a+RA groups (blue: HUVEC nuclei; indicated by white dashed box). Statistical analysis showing mean ± SD of adherent monocytes per region of interest (ROI) quantified via monocyte-wash-away assay with previous CD14-staining (CTR, C5a, and C5a+RA groups; N=6; scale bar: 40 µm). **(H)** Representative fluorescence images of human monocytes (green: CD14 labeled monocytes) adhesive to HUVEC monolayer of CTR, STEMI, and STEMI+RA groups (blue: HUVEC nuclei). Statistical analysis showing mean ± SD of adherent monocytes per region of interest (ROI) quantified via monocyte-wash-away assay with previous CD14-staining (CTR, STEMI, and STEMI+RA groups; N=6; scale bar: 40 µm). **(I)** Statistical analysis of nitric oxide (NO) concentrations in cell culture media supernatant. NO products were measured by chemiluminescence-based via NOA-280i for CTR, C5a, and C5a+RA groups (N=3) as well as for CTR, STEMI, and STEMI+RA groups (N=3). **(J)** Statistical evaluation of wound healing assays for CTR, C5a, and C5a+RA groups (N=7). Data showing the 24-h growth rate (µm/h) as described in the methods section as well as wound closure in % (*: CTR vs. C5a). **(K)** Statistical evaluation of wound healing assays for CTR, STEMI, and STEMI+RA groups (N=7). Data showing the 24-h growth rate (µm/h) as described in the methods section as well as wound closure in % (*: CTR vs. STEMI; +: CTR vs. STEMI+RA; #: STEMI vs. STEMI+RA). Groups: CTR: stimulation with standard cell culture media; C5a: cell culture media + 50 ng/mL of C5a; C5a+RA: cell culture media + 50 ng/mL of C5a + C5a-Receptor antagonist (PMX53; 1:1000). Serum groups: CTR: stimulation with cell culture media + 10 % serum of healthy donors STEMI: cell culture media + 10 % serum of STEMI patients; STEMI+RA: cell culture media + 10 % serum of STEMI patients + C5a-Receptor antagonist (PMX53; 1:1000). p-values: ****p<0.0001; ***p<0.001; **p<0.01; *p<0.05; ++p<0.01; +p<0.05; #p<0.05.

To investigate the impact of C5a on inflammatory processes of the vascular endothelium, we performed monocyte adhesion measurements and wound healing assays and tested the endothelial NO production. Adhesion forces between leukocytes and endothelial monolayers were measured using AFM-based single-cell force spectroscopy. **Figure 6C** showing exemplary force-distance curve of SCFS and Figure 6F showing exemplary picture of AFM cantilever with mounted monocyte.

Stimulation with C5a increased adhesion forces between monocytes and the HUVEC surface by 19% compared to control stimulation (CTR vs. C5a: 39.0 ± 15.0 µN vs. 46.7 ± 16.7 µN; p<0.001) (**Figure 6A**). Adhesion energy, calculated as the area under the curve (AUC) of the force-distance curves, increased after C5a stimulation by 59% (p<0.001) compared to controls (**Figure 6B**). This could not be prevented by administering C5aRA.

Stimulation with STEMI serum resulted in both higher adhesion forces (p<0.0001; **Figure 6D**) and higher adhesion energy (p<0.001; **Figure 6E**) compared to control conditions. However, adhesion energy was significantly decreased by 17% after C5aRA treatment (C5a vs. C5aRA: 0.19 ± 0.1 nJ vs. 0.16 ± 0.1 nJ; p<0.05) (**Figure 6E**). By quantifying adherent monocytes these results were confirmed. Both C5a (p<0.0001; **Figure 6G**) and stimulation with STEMI serum (p<0.0001; **Figure 6H**) enhanced the monocyte count per region of interest (ROI) compared to controls, which could not be prevented by treatment with C5aRA.

Stimulation with both C5a (CTR vs. C5a: 670 ± 102 µM vs. 386 ± 62 µM; p<0.01) and STEMI serum (CTR vs. C5a: 545 ± 49 µM vs. 361 ± 36 µM; p<0.01) resulted in reduced NO production compared to controls (**Figure 6I**). In both cases, treatment with C5aRA significantly improved NO production. Compared to C5a the administration of C5aRA led to a 59% higher NO concentration (p<0.05). In the STEMI group the treatment with C5aRA enhanced NO production by 40% (p<0.05) (**Figure 6I**).

To investigate the migration characteristics of HUVEC monolayers after C5a and stimulation with STEMI serum, wound healing assays were performed. The endothelial growth rate (µm/h) over 24 h was significantly reduced by 63% after C5a stimulation (CTR vs. C5a: 18.8 ± 10 µm/h vs. 7.1 ± 2 µm/h; p<0.01) (**Figure 6J**). C5aRA enhanced the growth rate by 25% compared to C5a stimulation (p<0.05). Wound closure (%/h) was compared among all groups. Both CTR and C5a+RA groups reached 50% wound closure after 4 h. Wound closure was significantly further advanced after 3.5 h in controls compared to those in the C5a stimulation group (p<0.05).

stimulation with STEMI serum decreased the growth rate by 83% compared to controls (CTR vs. STEMI: 17.2 ± 6 µm/h vs. 2.9 ± .06 µm/h; p<0.001) (**Figure 6K**). In controls, 50% of wound closure was reached after 4 h whereas in the STEMI+RA group 50% closure was achieved after 9 h. The STEMI+RA group showed a nonsignificant trend towards faster growth rate compared to STEMI group by 27%. However, wound closure after 9 h was significantly further advanced in the STEMI+RA group compared to stimulation with STEMI serum (p<0.05) (**Figure 6K**).

## 4 Discussion

The endothelial surface layers, endothelial cortex, and eGC are highly vulnerable to toxins and factors released during AMI, resulting in loss of their vasoprotective function (18, 20, 35). AMI-induced endothelial damage is multifactorial and complex, caused by a variety of immunologic factors present in AMI serum that are associated with cardiac mechanical stress, generalized vascular trauma, and inflammatory response (1, 35). During AMI, several innate immune pathways, including those of the complement system, are activated in the early steps of the inflammatory response to myocardial ischemia (1, 13). In this study, the effects of the complement anaphylatoxin C5a on the nanomechanical properties as well as on vascular surface function were studied in the context of AMI.

For this, serum derived from patients with a first onset of STEMI was used in an *in vitro* model to quantify the impact of elevated levels of the anaphylatoxin C5a on endothelial surface mechanics and endothelial function. Stimulation with both STEMI serum and with C5a only decreased eGC height and stiffness, indicating a C5a-mediated shedding of the eGC. In parallel, both stimulations increased cortical stiffness with a subsequent reduction in NO concentrations, enhanced monocyte adhesion to the endothelium, and decreased wound healing capacity, all indicating progressively developing endothelial dysfunction and vascular inflammation during AMI-induced C5a elevation.

Furthermore, we demonstrated that inhibiting the complement anaphylatoxin C5a using the C5aRA (PMX53) significantly reduced complement-induced vascular damage and enhanced vascular function. eGC degradation and cortical stiffening with subsequent endothelial dysfunction were both attenuated after administering C5aRA. Additionally, knock-out of the C5aR1 in mice correlated with these effects. Our findings demonstrate that the C5a:C5a-Receptor1 axis plays a central role in mediating vascular surface deterioration in the context of AMI.

During AMI, the main constituents of the eGC can be detected in patients’ blood. Syndecan-1 (36), heparan sulfate (12), and hyaluronan (37) have been identified as biomarkers for eGC damage during myocardial ischemia. This also applies to our data. In addition, our data demonstrate positive correlations between eGC component levels of syndecan-1, heparan sulfate, and hyaluronan, with levels of C5a indicating a possible interaction between loss of eGC components due to complement activation. Moreover, increased serum levels of eGC components are by no means unimportant: Jung et al. identified syndecan-1 as an independent predictor of 30-day mortality in cardiogenic shock and Wernly et al. showed that high levels of syndecan-1 are independently associated with 6-month mortality after myocardial infarction (36, 38). In the present study, mortality was not analyzed as an endpoint, but the positive correlation between elevated C5a and days until hospital discharge suggests that patients with higher C5a levels are more severely ill than patients with lower C5a levels. Indeed, correlations between enhanced complement activity and disease severity have been shown for SARS-CoV-2 infections (39), but have so far not been demonstrated in the context of AMI.

The effects between enhanced complement activity and disease severity are also reflected in the mechanical alterations of the vasculature: In the present study, the functional height of eGC was drastically reduced after incubating primary endothelial cells with sera derived from AMI patients. Intriguingly, the eGC height and stiffness incrementally decreased with rising levels of C5a (LOW vs. HIGH). In parallel, the cortical stiffness increased with rising C5a levels and was accompanied by a decrease in NO bioavailability, indicating progression of endothelial damage with progressing C5a release. There was no difference in NO availability between CTR and the LOW group, which may be due to small group size and the co-occurrence of outliers in the LOW group. Correlations between NO and C5a were therefore calculated excluding this group, although other correlations in this manuscript showed no different associations when the data of the LOW group were excluded.

The cortical stiffening accompanied by elevated levels of C5a combined with reduced NO production are most likely conferred by multiple pathways, but our data suggest an important contribution of the terminal complement pathway, especially C5a. C5a is associated with cardiovascular complications such as atherosclerosis and acute thrombosis (40). Furthermore, excessive quantities of C5a have been detected in patients with the acute coronary syndrome, advanced atherosclerosis, and myocardial infarction and have been associated with increased cardiovascular risk in patients with advanced atherosclerosis (41).

The reduction in eGC height and stiffness (shedding) as well as stiffening of the endothelial cortex were also shown for C5a stimulation alone, demonstrating the role C5a plays in endothelial impairment during AMI. Explanations of the mechanism underlying eGC shedding and subsequent cortical stiffening are embedded in the structure of the eGC, especially syndecan-1, which functions as a backbone of the eGC formation. Syndecan-1 is composed of an ectodomain as well as a transmembrane and a cytoplasmic domain (42). The cytoplasmic domain is directly associated with the actin cytoskeletal network of the cellular cortex via linker proteins, such as syntenin and synectin, allowing mechanotransduction from the endothelial surface to intercellular cortex (42). In response, syndecan-1 initiates cytoskeletal alignment and focal adhesion formation via activation of RhoA (43). In our study, baseline RhoA activity was significantly higher after C5a stimulation – and after AMI stimulation – displaying a direct link between eGC shedding, cortical actin alignment, and C5a stimulation. At this point, the C5aR1 plays an important mechanistic role. Kaida et al. demonstrated an enhanced conversion of RhoA-GDP to RhoA-GTP dependent on the C5a-C5aR1 signal (44). Activation of RhoA regulates cell stiffness via its downstream target Rho-associated kinase (ROCK), which modulates both actin/myosin-based cytoskeletal tension and cortical actin network formation (45). Thus, binding of C5a to the C5aR1 enhances activation of ROCK via RhoA conversion, leading to endothelial stiffening which ultimately results in eGC impairment. All these effects could at least be partly prevented by administering C5aR1A (PMX53) in our study.

Despite the influence of C5a on the endothelium, we should not neglect mentioning that the effects described here are not inevitably complement-dependent, but are, in any case, complement-related. AMI-induced shedding of the eGC with subsequent impairment of endothelial function might be caused by a variety of factors present in the patients’ sera which are associated with cardiac mechanical stress, generalized vascular trauma, and an inflammatory response (18). Those biomarkers and effectors of eGC degradation, are elevated and activated during AMI, including proinflammatory factors like interleukins (35), catecholamines (46), CRP (47) as well as matrix metalloproteinases (MMPs) (35). Furthermore, myocardial infarction and the following reperfusion of the occluded vessel results in a burst of free radical formation with increased generation of reactive oxygen species (ROS) (48). The increased oxidative stress and imbalanced levels of the production and accumulation of ROS not only enhances tissue damage of the myocardium, but also leads to degradation of eGC molecules (48, 49).

AMI immediately activates the sympathoadrenal system, which is associated with an excessive increase in circulating catecholamines, which, in turn, are associated with eGC damage (46). Furthermore, other immunogenic factors such as interleukins (IL) are activated during AMI. IL induce a complex network of proinflammatory cytokines via expression of integrins on leukocytes and endothelial cells. They regulate and initiate inflammatory responses that are associated with worse myocardial function, larger infarct extent, and more severe I/R injury in AMI (35, 50). Interestingly, excessive C5a levels can direct the production of cytokines such as tumor necrosis factor α and IL-6, which both induce heparanase expression with subsequent degradation of the eGC (51, 52). This might, in part, explain the mechanisms underlying the development of endothelial dysfunction under elevated C5a conditions. C-reactive protein (CRP) is also elevated in AMI sera. CRP is an early inflammatory biomarker associated with stiffening of the endothelial cell cortex, as shown in our data (47). Moreover, CRP can trigger activation of the complement system and lead to cardiovascular complications such as atherosclerosis and acute thrombosis mediated by generating excessive C5a (5). Furthermore, AMI serum contains a variety of MMPs, a group of zinc ion-dependent proteases that degrade collagen and proteoglycans (35, 53). MMPs play a pivotal role in the development of atherosclerosis and in post-myocardial infarction cardiac remodeling as well as in the development of adverse outcomes (53, 54). C5a upregulates the expression MMP-1 and MMP-9, thus contributing to the extracellular matrix and eGC degradation (55, 56). The eGC component syndecan-1 is shed by MMP isoforms MMP-2, MMP-9, and MMP-14 (57). The up-regulated MMP-9 (58), which is even amplified by C5a (5), may be responsible for eGC impairment in AMI through cleavage of syndecan-1.

Exposure of primary endothelial cells to AMI serum significantly decreased NO bioavailability, the hallmark of endothelial dysfunction (20), indicating the presence of additional inhibitors/mechanisms suppressing NO release, which are abundant in AMI serum. In addition, stimulation with C5a alone reduced NO production. This reflects the dose-dependent influence of C5a contained in the AMI serum on NO production by uncoupling the endothelial NO synthase (eNOS) (59). Reduction in NO bioavailability was prevented by administering C5aR1A (PMX53). This clearly shows an association of the C5a:C5a-Receptor axis with NO bioavailabilty. Similar effects could be shown for C5a activation of pig pulmonary endothelium, which altered NOS translation with subsequent endothelial dysfunction (60).

AMI and C5a stimulation both enhanced monocyte adhesion forces quantified by single-cell force spectroscopy and in monocyte adhesion assays. By using the single-cell force spectroscopy modality adhesion forces and adhesion energy can be quantified precisely between a single monocyte and endothelial surfaces and therefore the mechanical effects of C5a or STEMI serum stimulation on cell-cell surface interactions can be measured. Our data illustrate a change from a quiescent to an activated endothelial surface, resulting in a proinflammatory and prothrombotic state. Due to its position on the endothelial surface, the eGC mediates and regulates these leukocyte-endothelium interactions (61). The eGC has been recognized as an important structure during leukocyte recruitment and adhesion (27) with a proadhesive function, thus playing a crucial role during inflammatory processes (61). A functional and intact eGC lies atop of adhesion molecules such as vascular cellular adhesion molecule-1 (VCAM-1) and intercellular adhesion-molecule-1 (ICAM-1), which are needed for leukocyte-endothelial interaction. Unless the barrier formed by the eGC is compromised, leukocytes are ‘tip-toeing’ with their cytoskeletal protrusions on the eGC and barely reaching the adhesion molecules at the endothelial surface (27). The enhanced adhesion forces between monocytes and endothelial monolayers after C5a stimulation proves that shedding of the eGC favors the formation of bonds between binding partners on monocytes and endothelial surfaces. Furthermore, activation of RhoA/ROCK signaling, as demonstrated in the present study, was shown to contribute to lysophosphatidic acid receptor-4-induced stimulation of VCAM-1 expression (62). Thus, our findings are in line with the current state of knowledge as the interaction of C5a with endothelial cells has been demonstrated to upregulate cellular adhesion molecules (ICAM-1, VCAM-1), promoting infiltration of leukocytes to vessel walls and contributing to inflammation and atherosclerosis (5).

In the present study both, purified C5a stimulation as well as elevated levels of C5a in AMI patient’s sera, generate structural and mechanical changes on the endothelial surface, including the eGC and the cellular cortex via the C5a:C5a-Receptor1 axis. C5a activates RhoA, which accelerates endothelial stiffening via its downstream target ROCK. This causes eGC impairment, ultimately resulting in endothelial dysfunction with reduced NO bioavailability, enhanced endothelial surface activation, and decreased wound healing velocity. In parallel, a variation of other mechanistic processes is triggered by AMI, resulting in further damage to the endothelial surface and reducing vascular function. Our study confirms the important role of the C5a:C5a receptor1 axis in the development of vascular diseases and thus opens up new therapeutic approaches to improve patient health in the context of AMI. Future investigations should evaluate the role of C5a antagonism as a potential post-AMI treatment to counteract the development of endothelial dysfunction. In addition, influencing the C5a:C5a-Receptor1 axis might represent a novel approach to protect or restore the eGC in acute cardiac ischemia and prevent further development of endothelial dysfunction in the event of AMI.

## Data availability statement

The raw data supporting the conclusions of this article will be made available by the authors, without undue reservation.

## Ethics statement

The studies involving humans were approved by the ethics committee of the University of Luebeck (Case: 19-310). The studies were conducted in accordance with the local legislation and institutional requirements. The participants provided their written informed consent to participate in this study. The animal study was approved by Ministerium für Landwirtschaft, Energiewende, Umwelt und Ländliche Räume, Kiel, Germany (Case: 39.2_2020-08-20_Karsten). The study was conducted in accordance with the local legislation and institutional requirements.

## Author contributions

CV: Conceptualization, Data curation, Formal Analysis, Investigation, Methodology, Project administration, Resources, Software, Supervision, Validation, Visualization, Writing – original draft, Writing – review & editing. SL: Formal Analysis, Investigation, Methodology, Writing – review & editing. CH: Formal Analysis, Investigation, Methodology, Writing – review & editing. BR: Investigation, Writing – review & editing, Formal Analysis, Methodology. BF: Investigation, Writing – review & editing, Supervision, Validation. LN: Data curation, Investigation, Writing – review & editing. JW: Data curation, Supervision, Writing – review & editing. LB: Investigation, Methodology, Writing – review & editing. HN: Writing – review & editing, Formal Analysis, Investigation, Methodology. MK: Writing – review & editing, Investigation, Methodology. CK: Supervision, Writing – review & editing. KK: Conceptualization, Funding acquisition, Resources, Supervision, Validation, Writing – review & editing.
